# Recurrent Hemarthrosis following Resections of Benign Bone Tumors: A Case Report of Two Pediatric Cases

**DOI:** 10.1155/2021/5533636

**Published:** 2021-08-03

**Authors:** Ryan H. Barnes, Dawn Harter, Robert J. Esther, Ganesh V. Kamath, Anna D. Vergun

**Affiliations:** ^1^Department of Orthopaedics, University of North Carolina at Chapel Hill, Chapel Hill, North Carolina, USA; ^2^Department of Pathology and Laboratory Medicine, University of North Carolina at Chapel Hill, North Carolina, USA

## Abstract

*Introduction/Cases*. Two pediatric patients presenting with benign bone tumors of the distal femur at the level of the suprapatellar fat pad developed late onset recurrent knee hemarthrosis following surgical treatment of the lesions. A sinus tract from the intramedullary bone to the knee capsule was discovered in both patients during surgical exploration. Resection of the sinus tract and full closure of the knee capsule resulted in no further recurrence. *Conclusion.* Postoperative knee effusions in patients following resection near the distal femur could represent hemarthrosis that require additional workup and treatment. Resection of the sinus tract successfully treats the hemarthrosis.

## 1. Introduction

There are three fat pads at the knee: the anterior suprapatellar, posterior suprapatellar, and infrapatellar (Hoffa's) fat pads [1]. The normal anatomy of the suprapatellar fat pad is triangular in shape and is located between the posterior aspect of the quadriceps tendon, the anterior joint capsule, and the superior aspect of the patellar cartilage [2]. The suprapatellar pouch has been described to extend 4 cm proximal to the superior pole of the patella [3]. This complex anatomy can lead to a high rate of undiagnosed intra-articular involvement with fractures of the distal femur or proximal tibia. Hemarthrosis is described as bleeding into a joint and can occur from a variety of pathology including trauma, hemophilia, or medication side effects. In pediatric patients age 9 to 14, 70% of patients with traumatic knee hemarthrosis had an intra-articular injury, with 56% of patients not having an identifiable injury on plain radiograph [4]. A prior case report noted recurrent hemarthrosis in relation to a synovial hemangioma in a pediatric patient, ultimately requiring arthroscopic debridement and oral medication with eventual resolution of symptoms [5]. Another case report detailed spontaneous hemarthrosis 7 months following retrograde flexible nailing for a femur fracture secondary to erosion of the nail through knee joint capsule [6]. In adults, recurrent hemarthrosis is also a known, but rare, complication following total knee arthroplasty (TKA), occurring in 1-2% of cases [7, 8]. The prevalence of hemarthrosis in benign bone tumors is not currently well defined in the literature.

## 2. Case #1 Presentation

**Figure 1:**
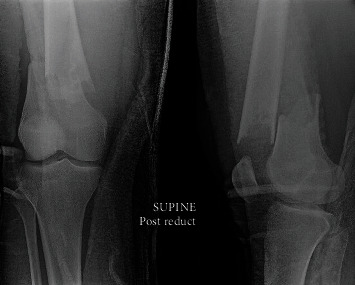
AP and lateral radiographs of the right knee demonstrating a pathologic fracture of the right distal femur. The lesion has characteristics consistent with nonossifying fibroma: lytic, cortically based lesion.

**Figure 2:**
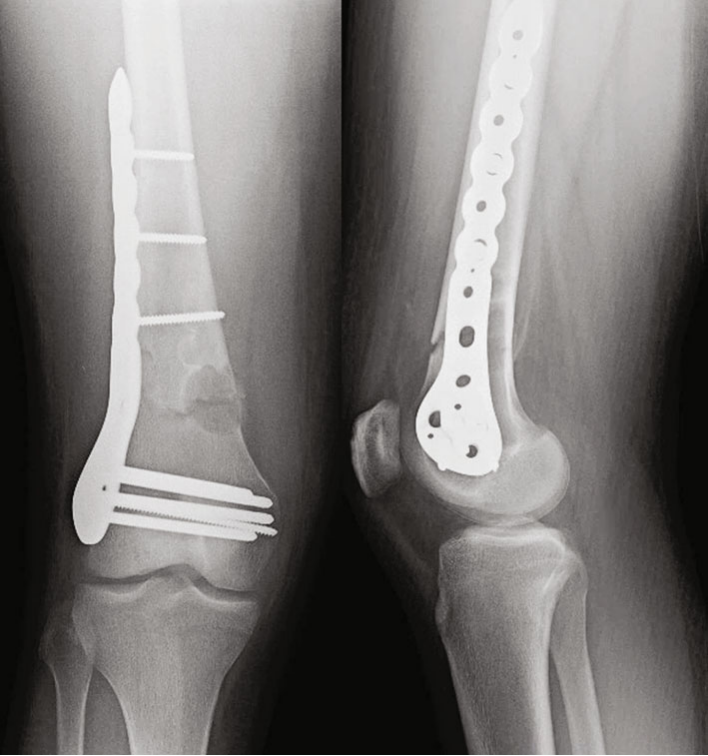
AP and lateral plain radiographs taken in clinic approximately 1 month postoperatively, demonstrating healing fracture.

**Figure 3:**
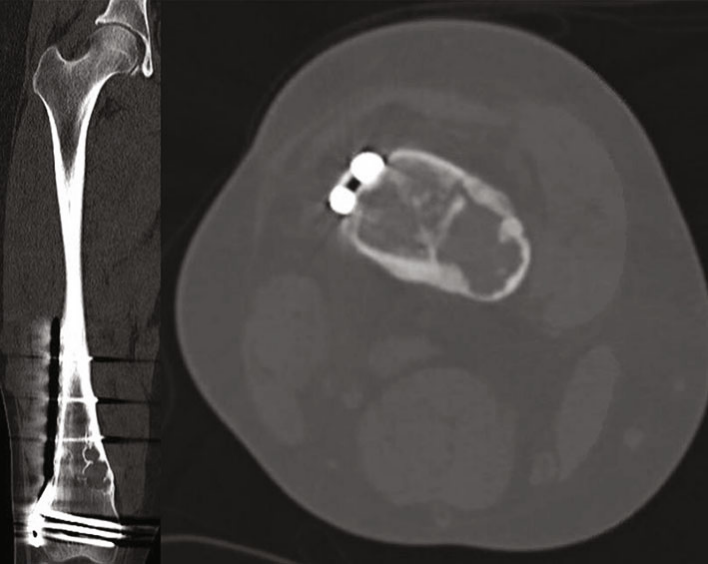
Computed tomography (CT) obtained 9 months status postopen reduction internal fixation of the distal femur showing a healed fracture without any evidence of any intra-articular hardware penetration.

**Figure 4:**
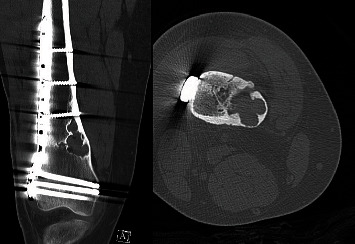
CT obtained 16 months postoperatively showing an anterior cortical defect approximately 7 cm proximal to the distal femur.

**Figure 5:**
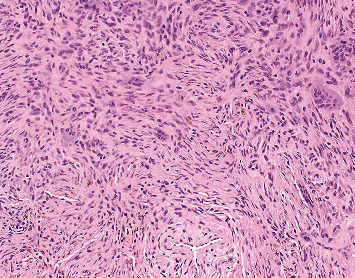
Pathology of nonossifying fibroma (magnification ×200). The lesion shows spindle cells in a storiform pattern with admixed multinucleated giant cells and hemosiderin-laden macrophages.

**Figure 6:**
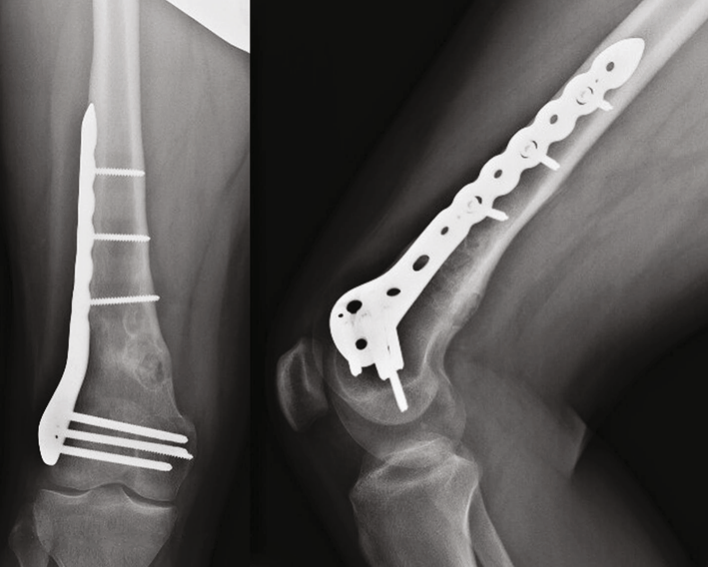
AP and lateral plain radiographs of the distal femur obtained at the final clinic appointment, 6 weeks postoperatively, following diagnostic arthroscopy, curettage, and bone grafting with completely healed fracture.

Case #1 is of a 16-year-old male with a history of attention-deficit disorder who presented to an outside pediatric emergency department after he experienced immediate right knee pain following a noncontact, twisting injury while participating in a football practice. At the outside emergency department, plain radiographs were obtained which demonstrated a pathologic fracture of the right distal femur (Figure 1). Prior to the injury, the patient denied having any night sweats or night pain but had noted vague knee pain which he attributed to a prior reported MCL injury. Given the benign appearance of the lesion, the fracture was definitively fixed using a lateral distal femoral locking (Figure 2). No curettage was performed. Approximately five months after surgery, the patient started experiencing increased pain around the knee which was initially attributed to hardware irritation. He continued to have pain, and a CT was ordered approximately nine months postoperatively to evaluate for union at the fracture site. The CT showed that all cortices had fully healed but the intramedullary lesion persisted. There were no hardware complications (Figure 3). 16 months postoperatively, he started to notice knee effusions after he had returned to playing football. Aspiration of the knee revealed a tense hemarthrosis. The hemarthrosis returned two days later, and a CT and MRI were obtained confirming a new hemarthrosis and a healed nonossifying fibroma (NOF) approximately 2.4 cm (width) by 2.1 cm (depth) and by 4.1 cm (length) proximal to the distal femoral. CT demonstrated an anterior cortical defect approximately 7 cm proximal to the distal femur (Figure 4). A second arthrocentesis was performed, yielding 30 mL of blood. Cell count, cultures, and crystals were unremarkable, and a coagulopathy workup was negative. Given his continued symptoms, the patient underwent a diagnostic arthroscopy, curettage, and bone grafting of the lesion. Arthroscopy showed persistent hemosiderin-tinged synovium throughout the knee with no evidence of screw penetration into the joint. However, the distal aspect of the plate was identified within the lateral gutter, and a sinus tract was discovered between the distal-most aspect of the NOF and the suprapatellar pouch. Specimen was collected for both frozen and permanent pathology, which was consistent with NOF (Figure 5). The sinus tract was resected, and careful closure of the capsule in the suprapatellar pouch was performed with absorbable suture. The lesion was then packed with cancellous allograft. He was last seen in clinic 6 weeks postoperatively, and he continued to do well with no return of symptoms, participating in activities as tolerated, with routine healing of the lesion (Figure 6). When he was contacted for permission to be included in this case study, 22 months following the last procedure, he had not had any recurrence of symptoms.

## 3. Case #2 Presentation

**Figure 7:**
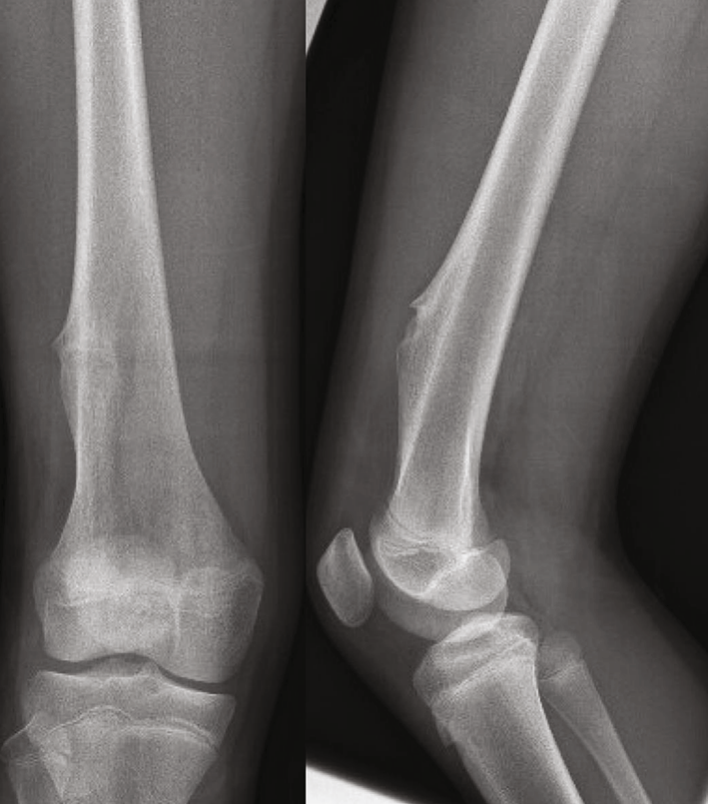
AP and lateral plain radiographs of the right distal femur demonstrate a sessile-appearing lesion on the anterolateral aspect of the distal femur consistent with osteochondroma.

**Figure 8:**
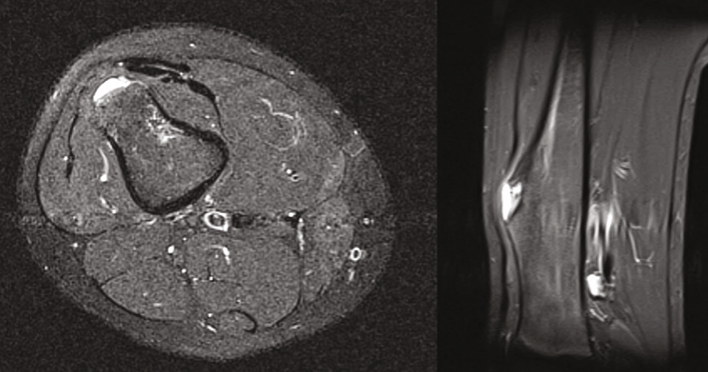
Magnetic resonance images (MRI) (machine settings: thickness 5.0 mm, fat-suppressed axial, and short-T1 inversion recovery (STIR) sagittal) demonstrating a distal femoral lesion consistent with likely osteochondroma.

**Figure 9:**
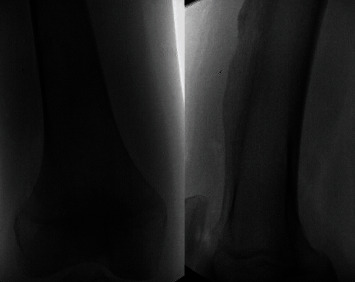
AP and lateral intraoperative fluoroscopic images showing further resection and smoothing of the distal femoral lesion.

**Figure 10:**
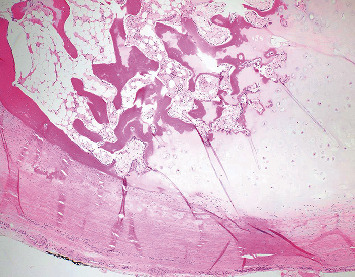
Pathology consistent with osteochondroma with cartilage cap (magnification ×40). The sessile lesion shows an intact smooth cartilaginous cap overlying bony trabeculae. The perichondrium that overlies the chondrocytes is continuous with the periosteum. The medullary cavity contains predominantly fat and fibrous tissue with minimal trilineage hematopoiesis. There are focal areas of the reactive bone with new bone formation.

Case #2 is of a 12-year-old female with an isolated symptomatic distal femoral osteochondroma approximately 10 cm proximal to the distal femur (Figure 7). An MRI was obtained to further evaluate the lesion and make final determinations regarding proceeding with resection (Figure 8). The lesion was surgically removed, and the cortical edges were smoothed with a high-speed burr. 10 weeks postoperatively, she noted swelling around the knee. 16 weeks postoperatively, the swelling had progressed and was limiting her mobility, and she presented to our emergency department with a large effusion. An arthrocentesis was performed, yielding 100 mL of blood. The fluid was sent for cell count, cultures, and crystals. All laboratory studies were negative. One week later, her symptoms recurred. A coagulopathy workup was negative. Approximately five months following initial surgery, the surgical site was explored, and a sinus tract was noted between the intramedullary bone of the anterior distal femoral osteochondroma resection site and the suprapatellar pouch. The bone lesion was resected further, the cortices were smoothed again, and bone wax was applied (Figure 9). The sinus tract leading to the suprapatellar pouch was resected, and the capsule was closed and plicated. The bone pathology remained consistent with an osteochondroma (Figure 10). She returned to clinic two weeks postoperatively with no return of symptoms. When she was called for permission to be included in this case study, 13 months following the last procedure, she had not had any recurrence of symptoms.

## 4. Discussion

These two cases represent recurrent hemarthrosis following resection of benign, symptomatic lesions. Existing literature reports rare occurrences of recurrent hemarthrosis in benign soft tissue tumors and following total knee arthroplasty. A single case report described recurrent hemarthrosis in the setting of a pediatric patient with synovial hemangioma requiring arthroscopic debridement [5]. Recurrent hemarthrosis has also been described following total knee arthroplasty, occurring in up to 2% of cases, with conservative management as the first line of treatment [8]. Further treatment is dependent upon the onset of symptoms and underlying cause with treatment varying from aspiration, cryotherapy, angiography, and revision arthroplasty. No case reports exist in the literature with either NOF or osteochondroma in either the pediatric or adult populations.

The cause of the recurrent hemarthrosis in these two cases is still unknown. However, both cases involved a benign bone lesion with an anterior distal femoral cortical defect near the suprapatellar pouch and a sinus tract connecting the intramedullary bone to the knee joint. Both lesions were over 6 cm from the joint line and were not originally considered intra-articular lesions. The suprapatellar pouch has been described to extend 4 cm proximal to the superior pole of the patella [3]. The lesions in both cases were near the joint but were not considered to be within the joint. Neither patient experienced significant swelling acutely postoperatively.

The authors speculate that the hemarthroses could have been prevented by inspection of the suprapatellar capsule and closure of any capsular defects during the index procedure. However, it is possible that there was no capsular defect during the index procedure. Rather, the defect was created from an inflammatory reaction from either the hardware in case #1 or from cortical irritation in case #2 which subsequently caused recurrent effusion. It is also possible that curettage and grafting of the lesion could have prevented the hemarthrosis.

## 5. Recommendations

Although rare, it is important to discuss with patients with distal femoral lesions with cortical defects near the suprapatellar pouch that could have recurrent hemarthrosis following resection. Educating patients for symptoms could prevent delayed diagnosis and prevent further sequela.

Although both patients were appropriately followed postoperatively and their symptoms addressed, successful treatment was delayed since there are no similar case reports, and the cause was not obvious until surgically explored. Another consideration is appropriate surgical treatment. Both patients had successful treatment with open procedures to address the capsular defect. Arthroscopic plication of the capsular rent could provide a sufficient treatment. In future cases, the authors have proposed the following workup: laboratory studies to rule out infection and coagulopathy, MRI to rule out further pathology, and diagnostic and therapeutic arthrocentesis. If the hemarthrosis recurs, the authors then recommend proceeding with surgical exploration for a sinus tract and resection of the tract with capsular plication. The authors recommend short-term follow-up with strict return precautions.

## Data Availability

Data is unavailable and cannot be released due to compliance with patient privacy.
